# Whey Protein Phospholipid Concentrate and Its Fractions as a Diet Intervention Enhance Bone Health and Alter the Gut Microbiome in Weanling Mice

**DOI:** 10.1096/fj.202502683R

**Published:** 2025-12-09

**Authors:** Mitchell T. Armstrong, Karen Antunes, Nathaniel B. Willis, Mark B. Meyer, Joseph F. Pierre, Gulustan Ozturk

**Affiliations:** ^1^ Department of Food Science University of Wisconsin‐Madison Madison Wisconsin USA; ^2^ Department of Nutritional Sciences University of Wisconsin‐Madison Madison Wisconsin USA

**Keywords:** bone, calcium transport, dairy, MFGM, microbiome, skeletal development, weanling mice, WPPC

## Abstract

Early post‐weaning growth is a critical window for bone development, and diet plays a central role in establishing peak bone mass in early life. Whey protein phospholipid concentrate (WPPC), a co‐product of whey protein isolate manufacturing, is enriched in bioactive lipids and proteins that may support bone development and calcium homeostasis. This study aimed to investigate the effects of WPPC and its protein‐ and lipid‐enriched fractions on bone development, gene expression, and gut microbiota in weanling mice. WPPC was fractionated using temperature‐dependent centrifugation, microfiltration, and ultrafiltration, yielding lipid‐ and protein‐rich components that were used to supplement isocaloric diets. Mice were fed either lipid fraction (Fat), protein fraction (Protein), or whole WPPC (WPPC) (10% by kcal; ~8.7% w/w) for 12 weeks. Compared to controls, the Protein and WPPC groups exhibited significantly increased femur length (4.44% and 4.01%, respectively), while the Fat group showed significantly higher bone mineral density (6.15%). Quantitative PCR of jejunal tissues revealed upregulation of calcium transporter genes (*Cldn2*, *Cldn12*, and *Pmca1*) in WPPC‐fed mice with no changes in vertebral bone markers of osteocyte differentiation. Circulating iFGF23 levels also remained unchanged, suggesting limited endocrine involvement. Gut microbiota analysis via 16S rRNA sequencing showed diet‐specific shifts, including increased *Akkermansia* and *Streptococcus* in the WPPC group and elevated *Lactobacillaceae* in the Protein and Fat groups. These findings demonstrate that WPPC and its enriched macronutrient fractions promote skeletal development and modulate calcium uptake and gut microbial composition, supporting their potential as functional ingredients for bone health applications in early life.

## Graphical abstract

Whey Protein Phospholipid Concentrate (WPPC), a dairy co‐product rich in bioactive lipids and proteins, was fractionated into lipid‐ and protein‐enriched components to evaluate their effects on skeletal development and gut health in weanling mice. Twelve‐week dietary intervention revealed that WPPC and its fractions enhanced bone mineral density and femur length, upregulated intestinal calcium‐transporter genes (Cldn2, Cldn12, Pmca1), and shifted gut microbiota toward beneficial taxa. Findings demonstrate WPPC's potential as a functional ingredient to support bone growth and calcium homeostasis.

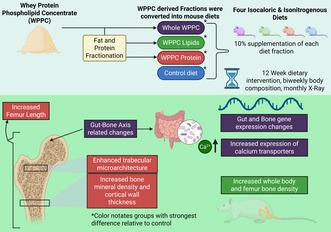

## 1 Introduction

Early‐life bone development is a critical window for achieving peak bone mass and minimizing the risk of osteoporosis later in life [1]. This process is governed by a complex interplay of genetic, hormonal, nutritional, and environmental factors, including the emerging influence of the gut microbiota [2, 3]. Nutritional strategies that enhance calcium absorption, regulate mineral metabolism, and shape the intestinal environment may support optimal skeletal outcomes during this critical growth period [2, 3, 4].

Dairy‐based foods are a rich source of bioavailable calcium, high‐quality protein, and bioactive lipids that may support these processes through both direct and indirect mechanisms [5, 6]. In recent years, attention has turned to underutilized dairy co‐products as sustainable and functional food ingredients. Among these, whey protein phospholipid concentrate (WPPC) has emerged as a promising candidate due to its unique composition of membrane‐rich lipids and high‐quality proteins [6]. WPPC is a complex dairy stream that contains proteins from both whey and the milk fat globule membrane (MFGM) proteins, as well as a rich array of phospholipids and neutral lipids [6, 7]. These components have been independently associated with various physiological benefits, including enhanced calcium absorption, improved gut barrier function, and modulation of the microbiome [4]. Despite its promising bioactivity, WPPC remains highly underutilized in human nutrition, with the majority of its industrial production—estimated to exceed 100 million pounds of powder in 2024—currently diverted to animal feed [6, 8]. One major limitation to WPPC's expanded use is its structural complexity [9]. Industrial processing involving ultrafiltration, microfiltration, and heat treatments produces tightly bound protein‐lipid aggregates that resist separation and functional characterization [8, 10]. As a result, the specific biological roles of its protein and lipid constituents, especially MFGM‐associated molecules, remain unclear. To better understand the specific contributions of WPPC components to health outcomes, we optimized fractionation strategies to subsequently assess the protein‐ and lipid‐enriched portions separately. To enable such investigation, a scalable, non‐solvent based, food grade fractionation method for isolating the protein and fat fractions from WPPC was ultimately developed to elucidate macronutrient‐specific effects.

While whey protein isolates [11], and specific peptides (e.g., glycomacropeptide) [5] have been shown to influence bone density and metabolic outcomes in mice, studies on dairy‐derived lipids remain limited and show mixed results depending on lipid class and experimental model [12, 13]. Moreover, no studies to date have explored WPPC or its fractions as dietary modulators of skeletal development, particularly in the context of the gut–bone axis—a rapidly growing field highlighting the interplay between intestinal function, microbial communities, and bone homeostasis.

In this study, the protein fraction was obtained using a microfiltration–ultrafiltration method developed by our group [14], while the lipid fraction was produced using a newly optimized, food‐grade, solvent‐free protocol designed specifically for this study. We characterized these fractions using proteomic and lipidomic mass spectrometry to confirm their compositional divergence from the original WPPC. These materials were then incorporated into isocaloric, nutrient‐matched diets and fed to weanling mice over 12 weeks. The experimental design included a whole WPPC group, a lipid fraction group (Fat), and a protein fraction group (Protein), compared to a macronutrient‐matched Control. Endpoints included bone mineral density (BMD), femur length, cortical microarchitecture, intestinal gene expression related to calcium transport, circulating levels of intact fibroblast growth factor 23 (iFGF23), and gut microbial composition.

## 2 Materials & Methods

### 2.1 Fractionation and Characterization of WPPC Proteins and Lipids

Over 175 gal of liquid WPPC were collected on the day of production from the Actus Nutrition processing facility in Wisconsin, USA. It was then transported back to the University of Wisconsin‐Madison under chilled conditions and frozen at −20°C for later processing. Filtration was conducted using custom filtration systems constructed in‐house. Membranes were sourced from Synder Filtration. Microfiltration (MF) was conducted with a 0.1 μm pore size, 12″ by 1.8″ spiral wound filtration membrane with 3.6 sq. ft. membrane area (SN: V0.1‐2B‐1212F). Ultrafiltration (UF) was conducted with a 30 kDa 12″ by 1.8″ spiral wound filtration membrane with 3.6 sq. ft. membrane area (SN: MK‐2B‐1812F). To achieve optimal pressures and flow rates, two GRI 115 V 60 Hz, 2 amp pumps (MN: 18650‐051S) were used in a 2‐pump cross‐flow system. Microfiltration was conducted with a 5 psi baseline pressure and a 12 psi boost pressure. Ultrafiltration was conducted with a 12 psi baseline pressure and a 20 psi boost pressure.

To produce the protein fraction, WPPC was diluted 1:2 with RO water prior to MF. The MF permeate, containing low molecular weight proteins, was then concentrated via UF to reduce water and proteins < 30 kDa [14]. The UF retentate, enriched in proteins, was freeze‐dried and stored at −80°C for later compositional analysis and dietary incorporation.

The fat fraction was generated from frozen WPPC by dilution (1:2 with RO water), followed by heating to 40°C and high‐speed agitation for 30 min. This mixture was then centrifuged at 7000 ***g*** for 30 min at 4°C. The upper solidified fat layer was separated from the aqueous phase and subjected to two successive washing steps with deionized water (1:2), each followed by warming to 40°C and centrifugation at 4°C. The final washed lipid fraction was stored at −80°C until use.

**FIGURE 1:**
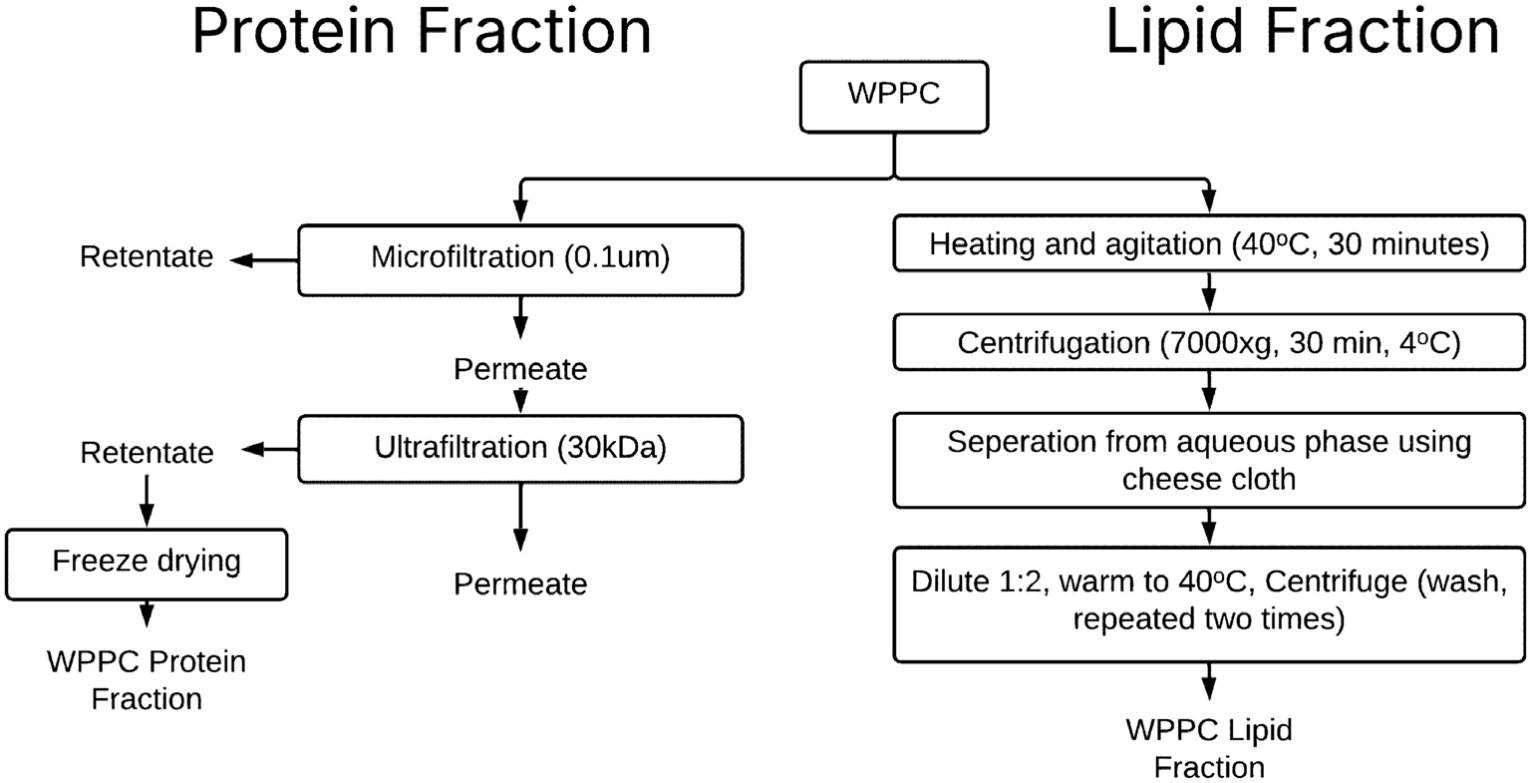
Fractionation and enrichment of WPPC lipids and proteins. Shown are filtration‐based and centrifugation‐based methods for the separation of protein and fat from WPPC. Filtration was two‐phase, with microfiltration (0.1 μm) followed by ultrafiltration (30 kDa) of the permeate (left). Lipid extraction was conducted using a 1:1 dilution of WPPC with heating, agitation, and cold centrifugation (right).

A full diagram of the filtration and fractionation workflow is shown in Figure 1.

### 2.2 Compositional Testing

Protein and fat in each fraction were measured by AOAC Official Methods (990.03, 2003.05). Simple sugars, including lactose, glucose, and galactose, were quantified using high‐performance liquid chromatography (HPLC).

### 2.3 Proteomic and Lipidomic Analysis

Proteomic and lipidomic analysis was performed at the University of Wisconsin Biotechnology Center Mass Spectrometry Core Facility. Protein pellets were isolated via methanol:chloroform:water extraction, washed with cold solvents, and resolubilized in 8 M urea/50 mM NH₄HCO₃ (pH 8.5). After BCA quantification, 35 μg protein per sample was reduced with DTT, alkylated with chloroacetamide, and digested with Trypsin/LysC. Digests were desalted (Pierce C18 tips), dried, and reconstituted in 0.1% formic acid. Peptides were separated by nanoLC and analyzed using an LTQ‐Orbitrap Elite mass spectrometer with a PepMap C18 capillary column. Survey scans (MS1) were acquired at 120 000 resolutions, followed by CID fragmentation of the top 30 precursor ions. MS/MS spectra were searched against the *Bos taurus* reference proteome using Scaffold software. Proteins were accepted at > 95% probability (≥ 1 peptide), and relative abundance was calculated based on normalized spectral counts.

Lipids were extracted using a modified Matyash method with PBS, methanol containing SPLASH LipidoMix internal standards, and methyl tert‐butyl ether (MTBE) [15]. Freeze‐dried WPPC (15.67 mg) or lipid fraction (16.67 mg) was homogenized (Qiagen TissueLyzer II) in a two‐step extraction procedure. After centrifugation (17 000 ***g***, 10 min, 4°C), the upper MTBE layer was collected, dried, and reconstituted in isopropanol. Process blanks, spiked blanks, and NIST SRM‐1950 plasma reference were included as quality controls. Samples were analyzed via UHPLC–MS and MS/MS in both positive and negative ion modes (Agilent QTOF), using standard gradients and internal mass calibrants. Lipids were identified using Lipid Annotator software and quantified as area under the curve (AUC); results are reported as relative abundance between sample types.

### 2.4 Animals and Experimental Design

All animal experiments were approved by the University of Wisconsin‐Madison Institutional Animal Care and Use Committee (protocol number A006568). Three‐week‐old male C57BL/6 mice (*n* = 36) were purchased from The Jackson Laboratory (Bar Harbor, ME) and housed in groups of three per cage under standard environmental conditions (25°C, 12‐h light/dark cycle) in a specific pathogen‐free facility. Upon arrival, animals were acclimated for 1 week with ad libitum access to standard chow and water. Mice were then randomly assigned to four experimental diet groups (*n* = 9 per group) with equivalent average body weights. The groups were designated as Control, Fat, Protein, and WPPC, corresponding to the dietary supplement received.

At 4 weeks of age, mice began a 12‐week dietary intervention. All diets were formulated to be isocaloric and matched for macronutrient content, with supplemental vitamin and mineral concentrations in excess of NRC requirements (Table 1, Figure S1). The non‐supplemented base diet was modeled after Teklad 2018 (Envigo). WPPC and its lipid and protein fractions were freeze‐dried and incorporated into custom chow by Research Diets International (New Brunswick, NJ). Macronutrient balance in the Protein and Fat diets was adjusted using added lard or whey protein isolate (WPI), respectively. All diets were gamma‐irradiated to ensure microbial safety following the addition of dairy‐derived fractions. Detailed formulation information is provided in Supporting Information. To control cage‐specific effects on the gut microbiome, bedding was mixed weekly within each experimental group.

**TABLE 1:** Mouse diet formulations.

Component (%w)	Base diet	Control group diet	Fat group diet (8.7% lipid fraction)	Protein group diet (8.7% protein fraction)	WPPC group diet (8.7% whole WPPC)
Protein	19.7	23.6	24.1	24.1	24.1
Carbohydrate	59	44.2	44.2	44.2	44.2
Fat	19	31.7	31.7	31.7	31.7
Lipid fraction	0	0	8.7	0	0
Protein fraction	0	0	0	8.7	0
Whole WPPC	0	0	0	0	8.7

*Note:* Values presented are the percentage calculated values for diet formulation, not measured values.

Throughout the intervention, dual‐energy X‐ray absorptiometry (DXA) scans were performed at baseline (Week 0) and at Weeks 4, 8, and 12 to assess femur bone length and bone mineral density (BMD). Body composition, including lean and fat mass, was measured biweekly (Weeks 0, 2, 4, 6, 8, 10, and 12) using quantitative nuclear magnetic resonance (NMR) spectroscopy (Minispec LF90II, Bruker, Karlsruhe, Germany) with daily phantom calibration. Body weights were recorded weekly using a digital analytical scale. Fecal samples were collected pre‐intervention and at the endpoint (Week 13) for microbiome analysis. At harvest, mice were anesthetized with isoflurane, and tissues and blood were collected. Blood was collected immediately after anesthetization via cardiac puncture. Tissues collected were stored at −80°C. Collected tissues are shown in Table S2.

### 2.5 Bone Morphology

Left femurs were harvested at the time of sacrifice and stored in phosphate‐buffered saline (PBS, pH 7.0) at −20°C until analysis. Micro‐computed tomography (micro‐CT) was performed using a MILabs CT‐UHR ultra‐high‐resolution scanner operated in step‐and‐shoot ultra‐focus mode. Imaging parameters included an exposure time of 85 ms, X‐ray tube voltage of 65 kVp, current of 0.13 mA, a rotation step angle of 0.10°, and 1 × 1 binning. Raw data were reconstructed using 20 μm isotropic voxel resolution. Image processing and analysis were conducted using Imalytics Preclinical software, following established micro‐CT bone imaging guidelines [16]. Scans were smoothed using a Gaussian filter (*σ* = 1.0 μm) to reduce image noise. Isolated femurs were cleaned of adjacent tissues digitally and segmented into two primary regions of interest (ROIs): trabecular and cortical compartments.

Trabecular bone analysis was conducted at the distal femur, defined as a 1 mm section starting at the growth plate and extending proximally. Cortical bone was analyzed using a 1 mm thick segment centered at the femur midpoint, defined as halfway between the femoral head and distal ridge. Bone segmentation was performed using a fixed threshold of 1400 arbitrary units (AU). Quantitative measurements included bone volume fraction, trabecular thickness, number, spacing, and cortical thickness. To determine femur length, the DXA scans were rendered in ImageJ.

Femur length was determined from DXA images, which were rendered and measured in ImageJ (NIH). Manual length measurements were conducted in triplicate by a blinded investigator to ensure accuracy and reduce bias. Bone mineral density (BMD) measurements were collected every 4 weeks beginning at baseline (Week 0) using dual‐energy X‐ray absorptiometry (DXA) with a PIXImus II densitometer (GE Lunar, Madison, WI). Mice were lightly anesthetized using isoflurane during all DXA measurements.

Whole‐body BMD was calculated by excluding the calvarium, scapulae, and the first few thoracic vertebrae. Femur BMD was determined using a rectangular region of interest (ROI) spanning from the distal femoral condyles to the femoral head. Instrument calibration was verified daily using a manufacturer‐provided skeletal phantom, following quality control protocols. Measurement of BMD followed a modified version of the method described by [17].

### 2.6 Analysis of Gut Microbiota

Jejunal and fecal samples were homogenized in 1 mL of extraction buffer [50 mM Tris (pH 7.4), 100 mM EDTA (pH 8.0), 400 mM NaCl, 0.5% SDS] supplemented with 20 μL of proteinase K (20 mg/mL) in 1.5 mL microcentrifuge tubes. To facilitate mechanical lysis, 0.1 mm diameter silica beads were added, and samples were disrupted using a Mini‐Beadbeater‐8 (BioSpec Products, Bartlesville, OK) for two 1‐min cycles. Lysates were incubated overnight at 55°C on a shaking platform. Genomic DNA was extracted using a phenol:chloroform:isoamyl alcohol method followed by ethanol precipitation. DNA was resuspended in nuclease‐free water and quantified using a NanoDrop spectrophotometer (ThermoFisher Scientific).

Extracted DNA was submitted to Novogene Co. Ltd. (Beijing, China) for library preparation and paired‐end sequencing of the V3–V4 hypervariable regions of the 16S rRNA gene using the Illumina MiSeq platform. Primers used were 341F (5′‐CCTACGGGNGGCWGCAG‐3′) and 805R (5′‐GACTACHVGGGTATCTAATCC‐3′).

Sequencing data were processed using QIIME2 (version 2024.10) for demultiplexing and denoising [17]. DADA2 was used for quality filtering, dereplication, and chimera removal, with a quality score threshold set at Q30 [18]. The resulting feature table included 1401 amplicon sequence variants (ASVs) with a mean sequence length of 419 bp. After filtering for low‐abundance and low‐prevalence features (< 10 counts in < 20% of samples), 170 features were retained. The final dataset included 2 129 482 total reads with a median of 44 364 reads per sample.

Taxonomic classification was performed using a naïve Bayes classifier trained on the Silva 138.2 NR99 SSU database for the 341F/805R region [19]. Phylogenetic trees were generated using the FastTree plugin within QIIME [20]. Total sum scaling was applied for normalization of feature counts to generate family‐level relative abundance plots.

All samples achieved sufficient sequencing depth, confirmed by rarefaction curves that reached richness saturation; therefore, no rarefaction was applied. Differential abundance analysis was conducted using linear discriminant analysis effect size [21]. Statistical comparisons were adjusted for multiple testing using the Benjamini‐Hochberg false discovery rate (FDR) method to control for type I errors.

### 2.7 Gene Expression

Total RNA was isolated from two tissues: the L5 vertebrae and the jejunal mucosa, to assess the effects of dietary treatments on bone‐ and calcium‐related gene expression. In the jejunal mucosa, mRNA expression levels of Fgf23 (fibroblast growth factor 23), *Pmca1* (plasma membrane calcium‐ATPase‐1), *Cldn2* (Claudin‐2), *Cldn12* (Claudin‐12), *Cabp‐9K* (calbindin‐D9k), *Ocln* (occludin), and *Bmp1* (bone morphogenic protein‐1) were measured. In the L5 vertebrae, the expression of key genes involved in bone formation and signaling—*Wnt* (β‐catenin pathway), *Osx* (Osterix or Sp7), *Runx2* (Runt‐related transcription factor 2), and *Ihh* (Indian hedgehog)—was assessed.

Tissue samples were homogenized in TRIzol reagent (Ambion, Austin, TX), and RNA was extracted using the TRIzol–chloroform method following the manufacturer's protocol. RNA purity and concentration were assessed using a NanoDrop Lite spectrophotometer (Thermo Scientific, Wilmington, DE). A total of 1.0 μg of RNA was reverse‐transcribed into complementary DNA (cDNA) using the Transcriptor First Strand cDNA Synthesis Kit (Roche, Indianapolis, IN).

Quantitative real‐time PCR (qRT‐PCR) was performed with the following cycling conditions: initial denaturation at 95°C for 10 min, followed by 45 cycles of denaturation at 95°C for 10 s, annealing at 55°C for 20 s, and extension at 60°C for 30 s. A final extension step at 55°C for 30 s and cooling at 40°C for 30 s was included. Primer sequences used for each gene target are provided in Table S1.

Gene expression levels were normalized to GAPDH, used as the reference housekeeping gene, and relative quantification was performed using the 2^−ΔΔCt^ method:

$$\begin{array}{c} \text{ΔΔCt}=\left[\mathrm{Ct}\;\left(\text{target gene}\right)–\mathrm{Ct}\;\left(\text{GAPDH}\right)\right]\;\text{test}\\ \;–\left[\mathrm{Ct}\;\left(\text{target gene}\right)–\mathrm{Ct}\;\left(\text{GAPDH}\right)\right]\_\text{control}. \end{array}$$

Data are presented as fold changes in mRNA expression relative to the control group.

### 2.8 Measurement of Plasma iFGF23

Blood was collected from anesthetized mice via cardiac puncture, and plasma was isolated using EDTA‐coated collection tubes as previously described [22]. Plasma levels of intact fibroblast growth factor 23 (iFGF23) were measured using a commercially available Mouse/Rat FGF23 (Intact) ELISA kit (Cat. #60‐6800, Quidel Corporation, San Diego, CA), following the manufacturer's instructions. All plasma samples were analyzed in duplicate to ensure accuracy and reproducibility.

### 2.9 Statistical Methods

All statistical analyses and graph generation were performed using GraphPad Prism (version 10). Longitudinal variables collected over the 12‐week study period—including body weight, whole‐body fat mass, lean mass, whole body BMD, femur length, and femur BMD—were analyzed using two‐way repeated measures ANOVA, followed by Tukey's post hoc test for multiple comparisons. Statistical significance was defined as a two‐tailed *p*‐value less than 0.05.

For single time‐point outcomes, including gene expression (qPCR), bone morphology (MicroCT), ELISA measurements (iFGF23), and blood chemistry data, one‐way ANOVA was employed with Tukey's post hoc test for multiple group comparisons. An alpha level of 0.05 was used to determine statistical significance.

## 3 Results

### 3.1 Produced Fraction Profile and Dietary Parameters

The microfiltration–ultrafiltration (MF–UF) method effectively extracted and concentrated protein from WPPC (Table 2). The initial microfiltration step yielded a permeate with negligible fat (0.05%) and low protein concentration (0.44% w/w), indicating that only 2.8% of the total WPPC protein was extracted at this stage. The MF retentate retained most of the protein and fat, confirming limited passage of macronutrients through the membrane. The subsequent ultrafiltration step successfully concentrated the protein in the UF retentate by more than four‐fold, reaching 2.05% (w/w), while the UF permeate contained near‐undetectable protein levels, demonstrating efficient protein retention. Importantly, the UF retentate, rich in protein and low in fat, was collected and freeze‐dried to generate the protein fraction used in the diet formulations.

**TABLE 2:** Composition of filtration fractions.

Material	Fat (%)	Protein (%)	Lactose (%)	Glucose (%)	Galactose (%)
WPPC	5.24 ± 0.002	18.0 ± 0.002	0.048 ± 0.02	0.0 ± 0.01	0.038 ± 0.01
MF retentate	3.86 ± 0.2	14.6 ± 0.53	0.014 ± 0.01	0 ± 0.01	0.20 ± 0.01
MF permeate	0.05 ± 0.003	0.44 ± 0.01	0.35 ± 0.02	0 ± 0.01	0.22 ± 0.01
UF retentate	0.07 ± 0.02	2.05 ± 0.65	0.043 ± 0.2	0 ± 0.01	0.26 ± 0.01
UF permeate	0.05 ± 0.01	< 0.05 ± 0.01	0.27 ± 0.01	0 ± 0.01	0.27 ± 0.01

*Note:* Values presented as mean ± SD. All samples shown are in liquid form collected during or immediately following filtration.

Carbohydrate analysis revealed that the original WPPC material contained lactose at 0.048% (w/w) and galactose at 0.038% (w/w). Lactose was present in low quantities across all fractions, with slightly higher levels in the MF and UF permeates (0.35% and 0.27%, respectively), consistent with the permeation of smaller sugar molecules through both filtration steps. Galactose levels followed a similar trend, with modest concentrations in MF (0.22%) and UF (0.27%) permeates, while glucose was largely undetectable in all fractions.

Following filtration, the MF permeate was enriched in lactose (0.35%) and galactose (0.22%), indicating successful passage of soluble mono‐ and disaccharides through the MF membrane. In contrast, the MF retentate retained minimal carbohydrate content, with lactose reduced to 0.014% and galactose increasing slightly to 0.20%. The UF permeate also contained modest amounts of lactose (0.27%) and galactose (0.27%). Glucose remained undetectable in all fractions (< 0.01%), consistent with its minimal presence in WPPC.

The freeze‐dried protein extract and the washed lipid fraction were enriched to 91.7% (w/w) and 86% (w/w), respectively, and were used in diet formulations (Table 3). In the final freeze‐dried diet formulations (Table 3), lactose remained present at low levels in all groups, ranging from 0.153% in the protein fraction to 0.192% in WPPC and 0.164% in the lipid fraction. These residual lactose levels were low enough to avoid confounding metabolic effects.

**TABLE 3:** Composition of diet fractions.

Diet fraction	Fat (%)	Protein (%)	Lactose (%)
WPPC	26.19 ± 0.01	58.75 ± 0.18	0.192 ± 0.01
Protein fraction	0.23 ± 0.16	91.65 ± 0.47	0.153 ± 0.01
Lipid fraction	86.00 ± 1.04	0.14 ± 0.03	0.164 ± 0.01

*Note:* Values presented as mean ± SD. WPPC and Protein fractions are freeze‐dried form. The lipid fraction was the final, washed, and undried product. Unaccounted products for WPPC and protein fraction were residual ash. Unaccounted product in the lipid fraction was a combination of ash and water.

### 3.2 Proteomic and Lipidomic Analysis of Whole WPPC and Enriched Protein Fraction

**FIGURE 2:**
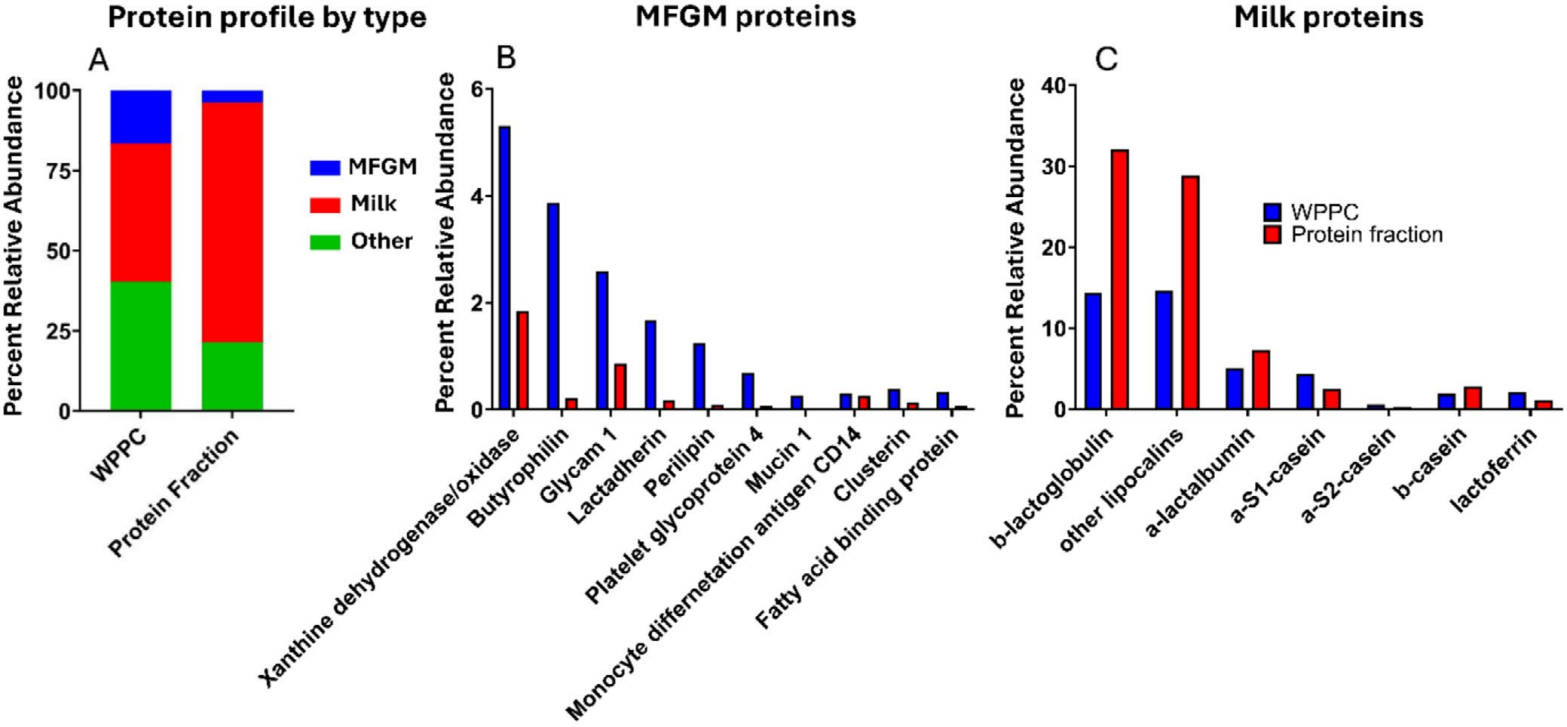
Proteomic analysis of whole WPPC and enriched protein fraction. Proteomics of WPPC and the produced protein fraction in which various protein abundances are expressed as a relative percentage of total protein. Proteins are divided into three groups: “MFGM proteins”, “Milk proteins”, and “Other proteins” (A). Relative abundance of specific MFGM proteins (B). Relative abundance of specific Milk proteins (C).

**FIGURE 3:**
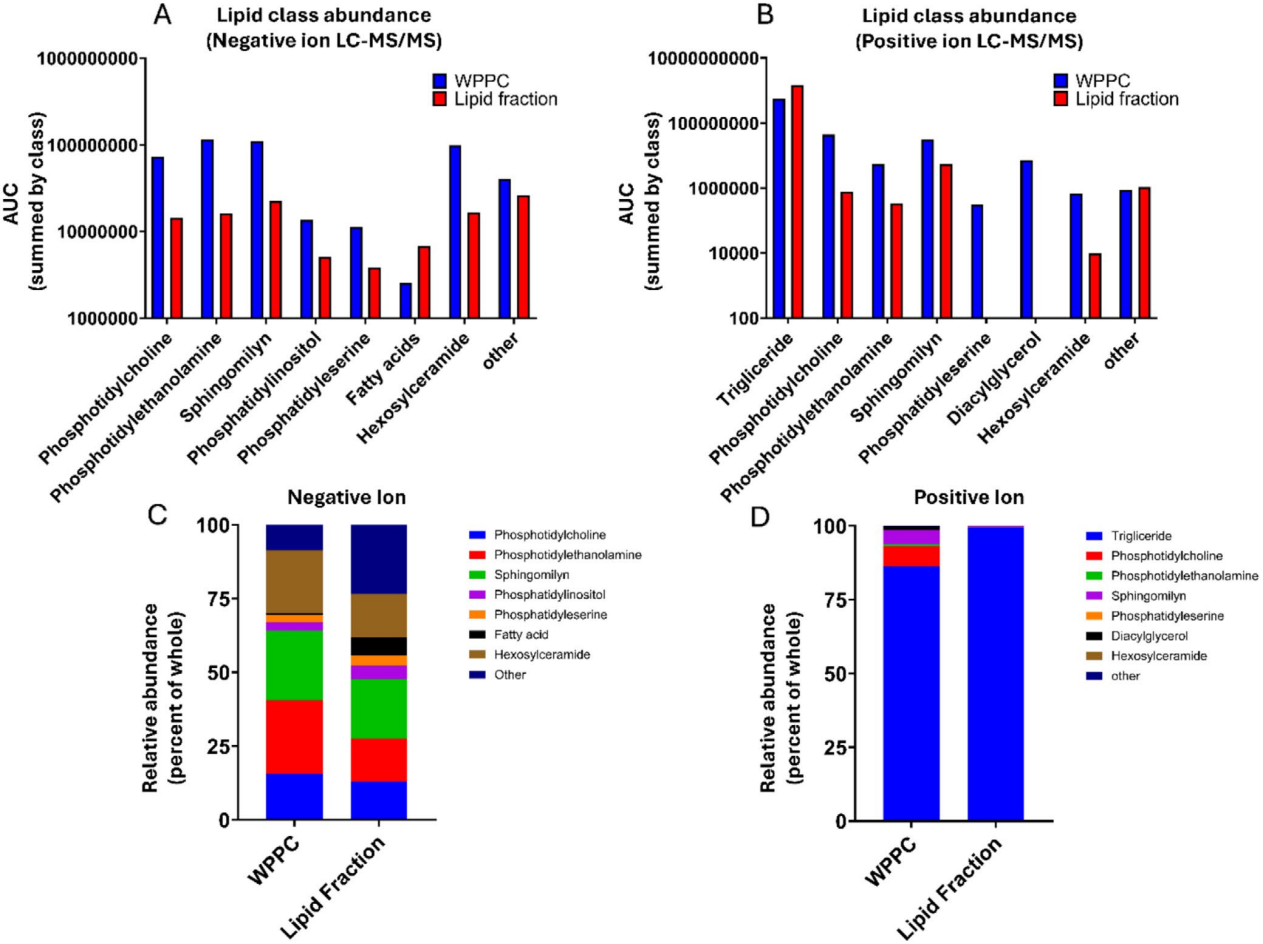
Lipidomic analysis of whole WPPC and enriched lipid fraction. Whole WPPC and the produced Lipid Fraction were analyzed using liquid chromatography mass spectrometry (LC–MS/MS) in both positive and negative ion modes. Shown is the percent relative abundance (AUC) of lipids by class. Results are shown in both percent of the whole (C, D) and as bar charts (A, B).

Mass spectrometry‐based proteomic and lipidomic analyses were conducted to characterize the composition of whole WPPC and the derived dietary fractions (Figures 2 and 3). The total number of proteins identified in WPPC was 343, while the protein‐enriched fraction contained 153 proteins. Protein classification revealed shifts in the proportional representation of major protein types (Figure 2A). The protein fraction was enriched in common milk proteins such as β‐lactoglobulin and α‐lactalbumin, with reduced levels of milk fat globule membrane (MFGM) proteins and other minor proteins (Figure 2B,C). Specifically, the relative abundance of β‐lactoglobulin increased substantially in the protein fraction compared to WPPC (Figure 2C). MFGM‐associated proteins such as xanthine dehydrogenase/oxidase, butyrophilin, and mucins were detected in both WPPC and the protein fraction, although at lower relative abundance in the latter.

In total, 427 lipids were detected in WPPC, while the lipid fraction retained 232 unique lipid species. Lipid class distribution differed markedly between WPPC and the lipid fraction, reflecting the separation of neutral and polar lipids during filtration. Lipidomic analysis in negative ion mode—optimized for polar lipids—showed that the lipid fraction preserved much of the phospholipid diversity present in WPPC, including phosphatidylcholine, phosphatidylethanolamine, and sphingomyelin (Figure 3A,C). However, there was a modest reduction in total phospholipid abundance and a relative increase in free fatty acids.

### 3.3 Dietary Intervention Had No Impact on Whole Body Weight, Lean Mass, and Body Fat Percent

**FIGURE 4:**
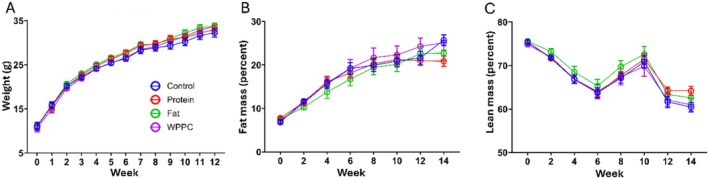
Dietary intervention had no impact on whole body weight, lean mass, and body fat percent. Changes in body weight during the 12‐week dietary intervention of mice fed the experimental Control, Protein, Fat, and WPPC diets (all *n* = 9) (A). Percent body fat changes over intervention (B). Percent lean mass changes over intervention (C). Two‐way ANOVA detected no significant differences between groups.

No significant differences in body weight or composition were observed among treatment groups throughout or after the 12‐week intervention (Figure 4). Food and water consumption were also similar across groups (Figure S2).

### 3.4 WPPC and Its Fractions Enhance Femur Length and Bone Mineral Density

**FIGURE 5:**
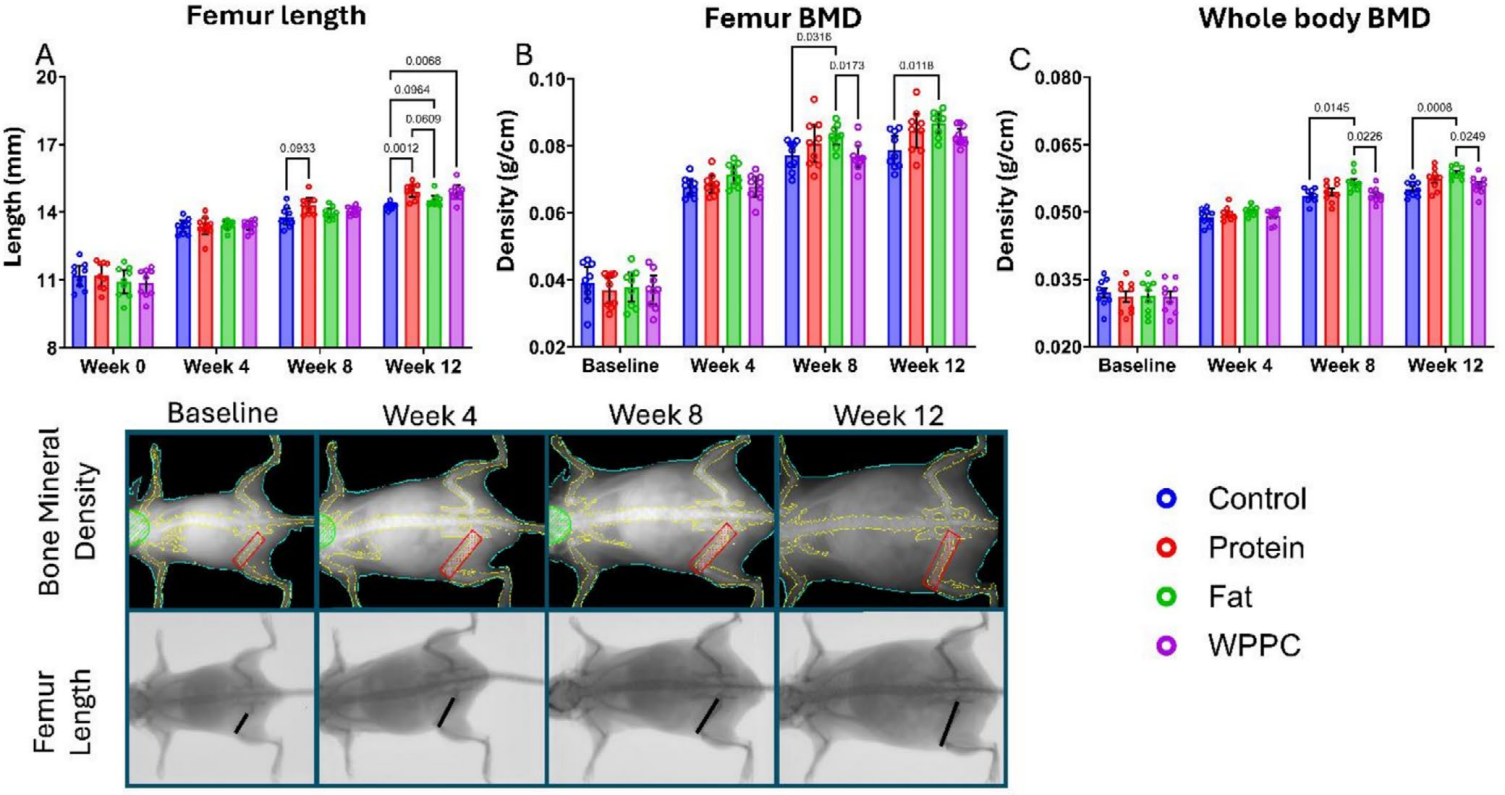
Whole WPPC and the Protein fraction increase femur length. The Fat fraction increases bone mineral density. Dietary effects on femur length (A), femur bone mineral density (B), and whole‐body bone mineral density (C) are shown for all experimental diet groups: Control, Protein, Fat, and WPPC, with *n* = 9 for each group at 4 separate timepoints by time of diet consumption. Values are expressed as means with their standard errors indicated by vertical bars. Data analyzed by two‐way ANOVA with Tukey's post hoc multiple comparison adjustment.

At baseline, no group differences in femur length were observed. After 12 weeks of dietary intervention, femur length increased significantly in the Protein and WPPC groups compared to Control, with a 4.24% and 4.20% increase, respectively (Figure 5A). The Fat group also showed qualitatively longer femurs, although this difference did not reach statistical significance. In terms of bone mineral density (BMD), the Fat group exhibited an increase of 9.34% (Figure 5B). These findings aligned with whole‐body BMD changes observed by DXA (Figure 5C), confirming the bone‐supportive effects of WPPC and its derived fractions.

### 3.5 WPPC and WPPC Fraction Supplementation Enhance Bone Microarchitecture

**FIGURE 6:**
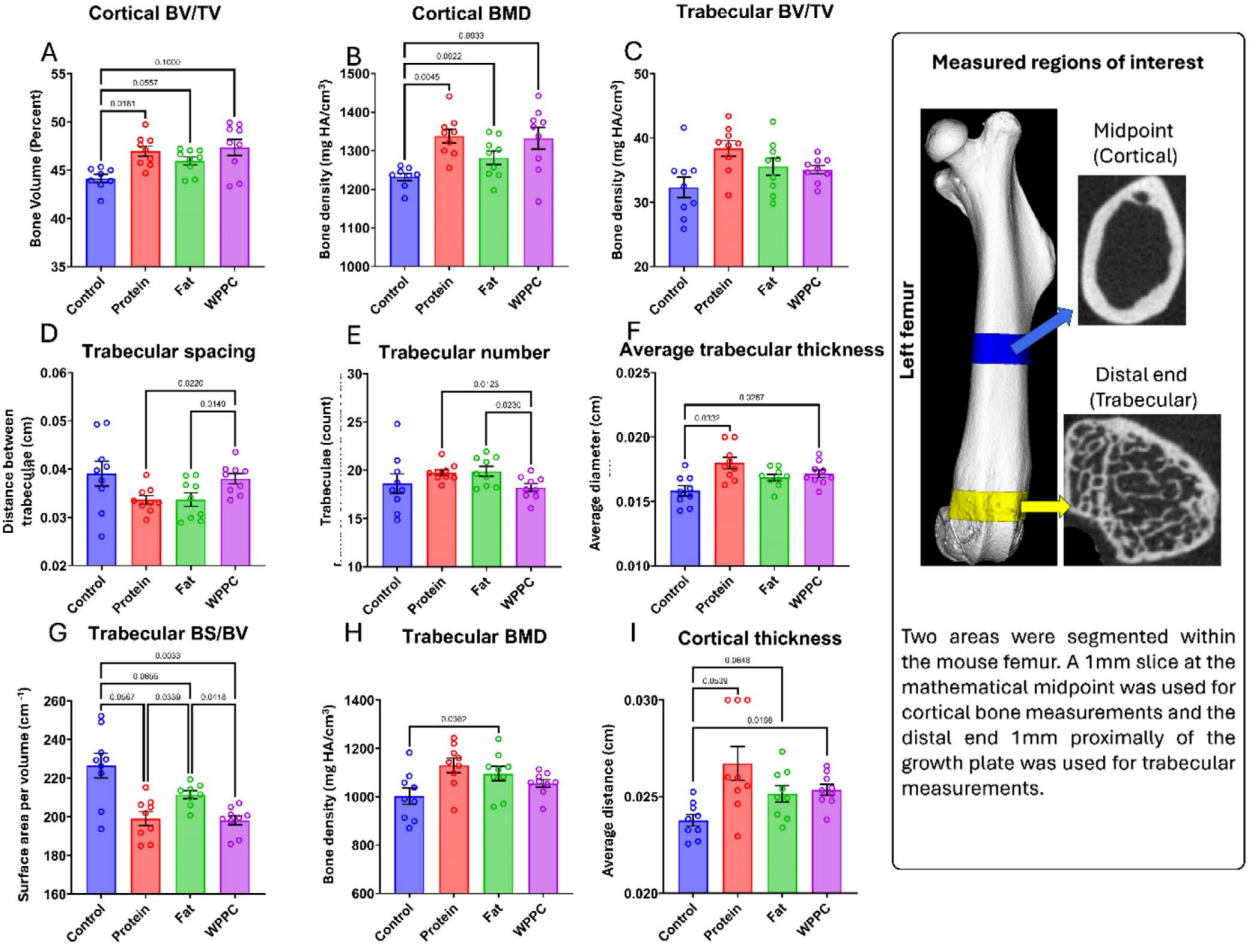
WPPC and WPPC fraction supplementation enhance bone microarchitecture. Quantitative parameters of the femur microarchitecture of all mice in each group (all *n* = 9). Shown metrics are: Cortical bone volume fraction (bone volume/total volume) (A), cortical bone mineral density (B), trabecular bone fraction (bone volume/total volume) (C), trabecular spacing (D), trabecular number (E), average trabecular thickness (F), trabecular surface area by volume (bone surface/bone volume) (G), trabecular bone mineral density (H), and cortical thickness (I). Trabecular bone measurements are taken from a segmented region of the femur starting at the epiphyseal growth plate extending one millimeter proximally (C–H). Cortical bone measurements are taken from a one‐millimeter slice centered at the mathematical midpoint of the femur (A, B, I).

Analysis revealed that all treatment groups (Fat, Protein, WPPC) exhibited significantly improved cortical bone thickness and trabecular density compared to control animals (Figure 6). Among the groups, the Protein group displayed the most pronounced enhancements in trabecular number, separation, and cortical thickness. WPPC and Fat groups also demonstrated significantly enhanced bone microarchitecture parameters. These differences showed partial concordance with the changes observed in two‐dimensional DXA measurements (Figure 5) and further support the role of WPPC components in promoting bone development and strength.

### 3.6 Expression of Jejunal and Vertebral Genes Associated With Calcium Homeostasis

**FIGURE 7:**
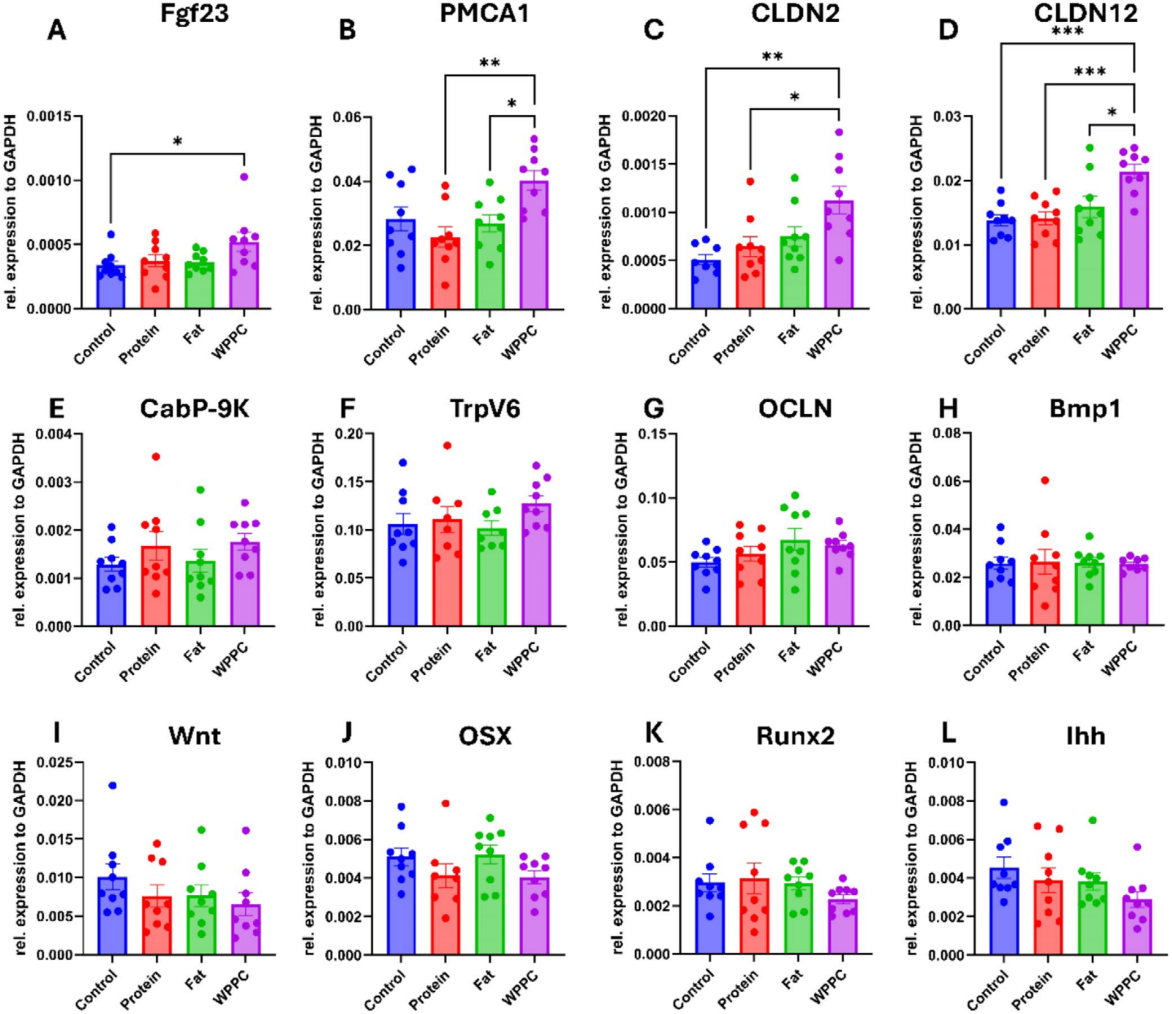
Expression of jejunal and vertebral genes associated with calcium homeostasis. Two tissue types were analyzed using quantitative PCR. Eight targets were measured in the jejunal mucosa scrapings (A–H), and a further four targets were measured in the L5 vertebrae (I–L). A one‐way ANOVA was used with Tukey's multiple comparisons. * notates *p* < 0.05, ** notates *p* < 0.01, *** notates *p* < 0.0001. All *n* = 9.

To explore mechanisms underlying the observed increases in femur length (Figure 5A), bone mineral density (Figure 5B,C), and microarchitecture (Figure 6), we assessed the expression of key genes involved in calcium transport and bone metabolism using quantitative PCR. In the jejunal mucosa, significant upregulation of key calcium transporter and tight junction genes (i.e., *Pmca1*, *Cldn2*, *Cldn12*, and *Fgf23*) was detected in the WPPC group. While TrpV6 and CaBP‐9 k were not statistically different across groups, they showed qualitative elevation in the Protein and WPPC groups compared with Control. In contrast, qPCR analysis of genes related to osteocyte differentiation in the L5 vertebrae (*Wnt, Osx, Runx2*, and *Ihh*) (Figure 7I–L) showed no differences between groups.

To assess whether dietary treatments altered systemic bone endocrine signaling, we measured circulating levels of iFGF23 using ELISA (Figure S2). Despite the observed upregulation of *Fgf23* gene expression in the jejunum, no statistically significant differences were found in plasma iFGF23 levels among groups. These findings suggest that the intestinal upregulation of *Fgf23* may reflect a localized, non‐secretory response or may not translate into elevated systemic iFGF23 under the conditions of this study.

### 3.7 Location Dependent Effects of WPPC and Derived Fractions on Gut Microbiota Relative Abundance

**FIGURE 8:**
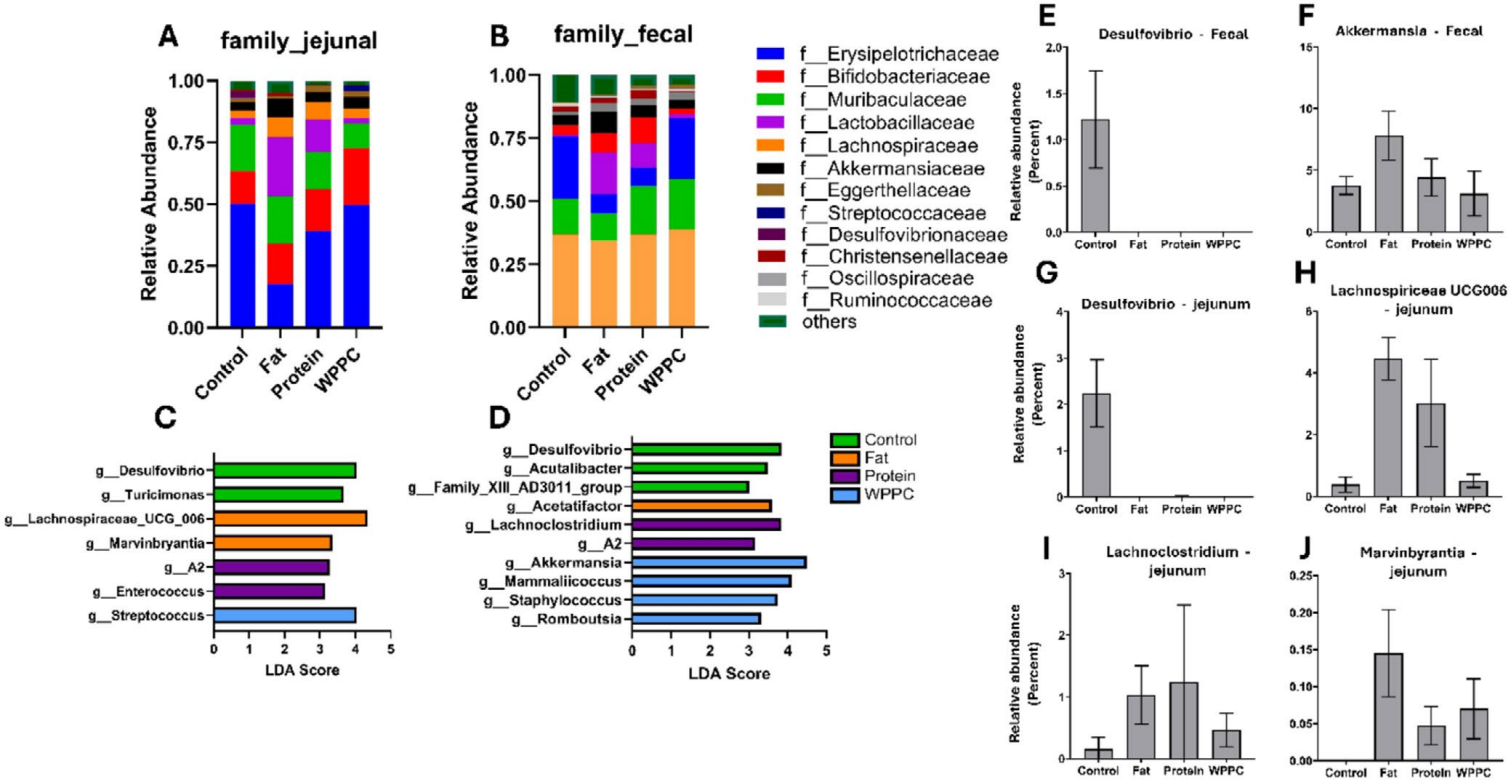
Jejunal and fecal microbiota relative abundance is altered by WPPC diets. (A) Colonic and (B) jejunal microbiota relative abundance at the family level. (C,D) LEfSe detected key genera most likely driving the differences between groups in the jejunal and colonic compartments, respectively. (E–J) Differential abundance of key genera by diet group. *n* = 5 for all groups.

Dietary inclusion of WPPC and its derived fractions resulted in distinct alterations in microbial communities across the gastrointestinal tract. At the family level, jejunal microbiota communities in all groups were dominated by *Erysipelotrichaceae*, *Bifidobacteriaceae*, and *Muribaculaceae* (Figure 8A,B). Notably, both the Fat and Protein groups exhibited a marked increase in *Lactobacillaceae* compared to Control, a trend that was consistent in the fecal microbiota profiles (Figure 8B).

Linear discriminant analysis effect size (LEfSe) identified differentially abundant genera that distinguished each diet group (Figure 8C,D). Genera of notable abundance in the Control group included *Desulfovibrio*, *Acutalibacter*, and *Turicimonas*, whereas *Marvinbryantia*, *Acetatifactor*, and *Lachnospiraceae UCG‐006* were more prevalent in the Fat group. The Protein group showed elevated levels of *Enterococcus* and *Lachnoclostridium*, while the WPPC group was characterized by increased *Streptococcus*, *Akkermansia*, and *Mammaliicoccus*.

Individual genera exhibiting significant changes by diet group and intestinal location are highlighted in Figure 8E–J. For instance, *Desulfovibrio* abundance was highest in fecal samples from Control animals, while *Lachnospiraceae UCG‐006* and *Marvinbryantia* were elevated in jejunal samples of the Fat group (Figure 8F,H,J). Higher relative abundance of *Lachnoclostridium* was observed in the jejunum of the Protein group (Figure 8I), whereas *Akkermansia* showed higher fecal abundance in WPPC‐fed animals (Figure 8F). These findings underscore the distinct and region‐specific modulatory effects of WPPC fractions on the gut microbiota.

## 4 Discussion

This study provides compelling evidence that whey protein phospholipid concentrate (WPPC) and its isolated lipid and protein fractions contribute to improved skeletal outcomes in weanling mice through both direct and indirect mechanisms. The observed increases in femur length and bone mineral density (BMD) suggest that WPPC is more than a simple macronutrient source—it contains bioactive components capable of modulating growth physiology.

This study provides a comprehensive physiological analysis of WPPC in a nutritional intervention model, with a focus on bone development. By conducting a 12‐week dietary intervention beginning in 3‐week‐old mice, a critical window for peak bone mass accrual—we captured skeletal and gut‐related adaptations relevant to adolescent growth temporally [2]. Across experimental groups, diets were matched for calories and macronutrients, with only ~10% of the total formulation differing by the inclusion of WPPC or its derived lipid or protein fractions.

Although all animals maintained normal growth trajectories and whole‐body composition, we observed that the WPPC and Protein groups exhibited significantly longer femurs, and the WPPC and Fat groups had increased BMD. These findings were accompanied by variable upregulation of intestinal calcium transporter transcripts, such as *Cldn2*, *Cldn12*, and *Pmca1*, as well as microbiota relative abundance shifts in the jejunum and colon. Together, these findings suggest that WPPC and its fractions may act through both gastrointestinal and microbiome‐mediated pathways to promote bone development.

Critically, the omics‐based characterization of the protein and lipid fractions revealed that the MFGM enrichment in both was lower than expected. Proteomic analyses showed that only ~4% of proteins in the protein fraction were attributable to MFGM. The lipid fraction was primarily composed of triglycerides, rather than polar phospholipids and glycosphingolipids, which are typically enriched in MFGM [6, 23]. This compositional shift likely reflects limitations in the fractionation method, including heat‐induced aggregation and the 0.1 μm membrane cutoff used during tangential flow filtration, which favors smaller whey proteins and may exclude larger protein‐lipid MFGM aggregates [7, 8, 10]. Nevertheless, the fractions retained physiological activity, supporting the possibility that non‐MFGM components of WPPC also contribute to its bone‐supportive effects. Similar results with other dairy‐based diet interventions have been reported [13, 24].

Analysis of trabecular and cortical bone architecture further supported the beneficial effects of WPPC and its fractions (Figure 6). Trabecular number, thickness, and spacing, all metrics linked to fracture risk and mechanical integrity [25], were improved in the WPPC and Protein groups. Cortical bone parameters also improved, particularly in the WPPC‐fed mice. These structural differences suggest that both trabecular and cortical compartments are sensitive to dietary modulation by dairy‐derived components. Interestingly, the Protein group, which was relatively enriched in common whey proteins such as β‐lactoglobulin, showed robust effects on femur length and microarchitecture—often exceeding those of the WPPC group. This suggests a strong osteogenic role for whey proteins specifically, consistent with prior studies linking dairy protein intake to bone mass accrual via IGF‐1 and calcium absorption pathways [26, 27].

At the intestinal level, the upregulation of calcium transporter genes in the WPPC and fraction groups suggests enhanced epithelial transport of calcium—likely driving the increased bone mineral deposition (Figure 7B–D). While osteocyte‐related gene expression did not vary substantially, previous work has suggested that small shifts in calcium absorption can have downstream effects on bone homeostasis, especially in rapidly growing animals [28]. As seen in other dietary intervention models, changes in epithelial transport (e.g., *Cldn12*, *Pmca1*) rather than osteoblast differentiation often precede skeletal improvements [1, 29]. The transcriptional profile observed in WPPC‐fed mice is consistent with established mechanisms of intestinal calcium absorption. Claudin‐2 and claudin‐12 form calcium‐permeable tight‐junction channels that mediate paracellular Ca^2+^ flux across the intestinal epithelium [30, 31, 32], and mice lacking these claudins exhibit impaired intestinal calcium uptake and reduced serum calcium, underscoring their functional significance [30]. In parallel, PMCA1 serves as the principal basolateral Ca^2+^ extrusion pump in enterocytes, supporting the transcellular arm of absorption [32]. The simultaneous upregulation of Cldn2, Cldn12, and Pmca1 therefore suggests a coordinated enhancement of both passive (paracellular) and active (transcellular) transport pathways, consistent with the greater BMD observed in the WPPC and Fat groups. Although direct measures of calcium flux were not included in this study, these results align closely with prior evidence linking these transcripts to improved calcium absorption and bone mineralization. Future mechanistic work using stable calcium‐isotope tracing and circulating bone‐turnover biomarkers (osteocalcin, CTX, ALP) will further confirm these functional effects. Additionally, there were no significant differences in circulating iFGF23 levels across groups (Figure S2). These findings align with prior work showing stable FGF23 expression in growing animals fed phosphate‐balanced diets [33, 34]. As FGF23 is strongly regulated by phosphate, vitamin D, and inflammation [35, 36], and our diets were phosphate‐matched, these results were expected. Importantly, while WPPC did not affect systemic FGF23 levels, it did significantly increase jejunal expression of calcium transporters. This supports the hypothesis that WPPC promotes bone mineralization via local intestinal mechanisms, independent of the endocrine system.

This study also captured significant modulation of the gut microbiota, with WPPC, Protein, and Fat groups each showing shifts in microbial taxa with known links to bone metabolism. For example, *Lachnoclostridium* and *Marvinbryantia*, genera linked to bone morphogenic proteins (BMPs) in recent genome‐wide microbiome studies [37], were increased in treated groups. Additionally, reductions in *Desulfovibrio*, a pro‐inflammatory genus associated with gut barrier disruption, were observed across groups, suggesting improved gut health [38]. There was a WPPC‐dependent increase in the relative abundance of SCFA‐producing families such as *Lachnospiraceae*, *Akkermansiaceae*, and *Bifidobacteriaceae*, which have a metabolic proclivity toward glycoproteins [14, 39], known components of WPPC [6]. These taxa may contribute to both calcium absorption and systemic anti‐inflammatory effects, factors known to affect bone remodeling [40, 41]. These specific taxa abundance changes echo previous findings where WPPC supplementation protected against cognitive decline by modulating microbial and lipidomic profiles [42, 43], suggesting WPPC‐induced gut microbiota community alterations may offer additional utility beyond the gut‐bone axis in combating the detrimental effects of aging.

The present study offers robust evidence in support of WPPC as a novel dairy product that may support the optimization of skeletal growth in a key developmental window. Despite these promising findings, the inability to fully isolate MFGM from WPPC remains a current limitation. While these data clearly show physiological benefits, the exact bioactive constituents remain difficult to pinpoint. Future work will involve enhanced fractionation techniques, including but not limited to gradient ultracentrifugation, immunoaffinity isolation, and gentle protease treatment to recover functional membrane proteins and polar lipids. Combining these efforts with advanced multi‐omics approaches will allow a more precise mapping of dietary components to physiological outcomes.

This study was intentionally designed as a first‐phase, mechanistic investigation to define the physiological effects and pathways by which WPPC and its fractions influence bone development and intestinal function. To minimize biological variability and isolate mechanistic responses, male weanling mice were selected as a controlled model of post‐weaning skeletal growth. Building on these findings, future studies will employ female, aged, and surgically induced osteoporotic models to evaluate whether WPPC and its fractions confer comparable skeletal and intestinal benefits under conditions of estrogen deficiency or bone loss. These investigations will expand upon the foundational evidence presented here and further define the translational potential of WPPC as a functional dietary ingredient for both pediatric and aging populations.

In conclusion, WPPC and its protein and lipid fractions improved skeletal health, altered calcium transport gene expression, and modulated gut microbiota relative abundance in mice during a critical developmental window. Although MFGM enrichment was lower than expected, our findings demonstrate that the underutilized WPPC‐derived bioactive components hold promise as nutritional interventions to support bone development. These results lay foundational groundwork for future research into functional dairy ingredients and their therapeutic applications in both pediatric and aging populations.

## Author Contributions

Conceptualization (Gulustan Ozturk, Joseph F. Pierre); Supervision (Gulustan Ozturk, Joseph F. Pierre); Project administration (Gulustan Ozturk), Data acquisition (Mitchell T. Armstrong, Karen Antunes, Nathaniel B. Willis, Mark B. Meyer, Joseph F. Pierre, Gulustan Ozturk); data analysis, statistical analysis, and interpretation (Mitchell T. Armstrong, Karen Antunes, Nathaniel B. Willis, Mark B. Meyer, Joseph F. Pierre, Gulustan Ozturk); Writing – original draft: (Mitchell T. Armstrong), Writing – review and editing (Mitchell T. Armstrong, Karen Antunes, Nathaniel B. Willis, Mark B. Meyer, Joseph F. Pierre, Gulustan Ozturk); obtaining funding (Gulustan Ozturk). All authors read and approved the manuscript.

## Funding

UW‐Madison‐Dairy Innovation Hub and National Institutes of Health grant T32 DK007665.

## Conflicts of Interest

The authors declare no conflicts of interest.

## Supporting information


**Table S1:** PCR primers used.
**Table S2:** Full diet formulation.
**Table S3:** Organ and tissue weights and lengths.
**Figure S1:** Food and water consumption.
**Figure S2:** Intact FGF23 serum levels.

## Data Availability

All data are provided in the article or Supporting Information.
