# Supplementary material for: Whey Protein Phospholipid Concentrate and Its Fractions as a Diet Intervention Enhance Bone Health and Alter the Gut Microbiome in Weanling Mice
Source: FASEB J. 2025 Dec 9;39(23):e71260. doi: 10.1096/fj.202502683R (PMC12687760; doi:10.1096/fj.202502683R)
Supplement: Supplementary file 1 — Table S1: PCR primers used. Table S2: Full diet formulation. Table S3: Organ and tissue weights and lengths. Figure S1: Food and water consumption. Figure S2: Intact FGF23 serum levels. [file FSB2-39-e71260-s001.docx]

Supplemental Figures and Tables

Supplementary Table 1: PCR primers used

| Supplemental table 1. Primers for q-RTPCR | | |
| --- | --- | --- |
| Primer | FWD | RV |
| GAPDH | 5’ - AGA CAA AAT GGT GAA GGT CG - 3’ | 5’ - AAT CTC CAC TTT GCC ACT G – 3' |
| Fgf23 | 5’ - AGG CTG AAC TCT GAA ATG TG – 3’ | 5’ - GTG CCT GCA AGT CGA AG – 3' |
| ATP2b1/Pmca1 | 5’ - GCT TTT TAC CCC ACC TCT AA – 3' | 5’ - GGA TCC AAG ATC TTT AAC AGC – 3' |
| Cldn2 | 5’ - AAG AAG GCT CAG AGT TAC AG – 3' | 5’ - GCT GTG GTG ATA AGG GAT TT – 3' |
| Cldn12 | 5’ - GGA GAA ACG CTG ATT ACC T – 3' | 5’ - CGT GTA AAT CGT CAG GTT CT – 3' |
| S100g/CabP-9K | 5’ - CGA TGG AGA AGT TAG TTA CGA – 3' | 5’ - CTT CCT GAC TTG TTC ATT GTG – 3' |
| Trpv6 | 5’ - AGG ACT CTC TGG GAA ATA CA – 3' | 5’ - AGG TGT TGG AAC ATC ACA AT – 3' |
| Ocln | 5’ - ACT GGG TCA GGG AAT ATC CA – 3' | 5’ - TCA GCA GCA GCC ATG TAC TC - 3’ |
| Bmp1 | 5’ - GTA TCC CAA TGG CTA CTC TG – 3' | 5’ - CTC CAC ATA GTC ATA CCA GC – 3' |
| Wnt3a | 5’ - GAT ACC TCT TAG TGC TCT GC – 3' | 5’ - CAT GAT CTC CAC GTA GTT CC – 3' |
| Osx/Sp7 | 5’ - CGA GTC AAG AGT AGG ATT GT – 3' | 5’ - TGT TTG ACG CCA TAG TCC – 3' |
| Runx2 | 5’ - CAG TCC ATG CAG GAA TAT TTA AG – 3' | 5’ - TGT TTG ACG CCA TAG TCC – 3' |
| Ihh | 5’ - CTA GAG AGA CAG GGC CTA TT – 3' | 5’ - TTG CGT GGT AGA TTC TGA AA – 3' |

Supplementary table 2: Full diet formulation

| **Diet** | **Control** |  | **Fat Fraction** |  | **Protein Fraction** |  | **WPPC** |  |
| --- | --- | --- | --- | --- | --- | --- | --- | --- |
| **Ingredient** | gm |  | gm |  | gm |  | gm |  |
| Casein | 240 |  | 240 |  | 240 |  | 240 |  |
| Whey Protein Isolate  (Alacen 895) | 119.5 |  | 119.3 |  | 0 |  | 42.9 |  |
| L-Cystine | 4 |  | 4 |  | 4 |  | 4 |  |
| Corn Starch | 313 |  | 313 |  | 313.6 |  | 313.4 |  |
| Maltodextrin | 110 |  | 110 |  | 110 |  | 110 |  |
| Dextrose | 150 |  | 150 |  | 150 |  | 150 |  |
| Sucrose | 3.65 |  | 3.65 |  | 3.65 |  | 3.65 |  |
| Cellulose | 135 |  | 135 |  | 135 |  | 135 |  |
| Inulin | 33.8 |  | 33.8 |  | 33.8 |  | 33.8 |  |
| Soybean Oil | 82 |  | 82 |  | 82 |  | 82 |  |
| Lard | 103.3 |  | 0 |  | 103.5 |  | 72.2 |  |
| t-BHQ | 0.036 |  | 0.036 |  | 0.036 |  | 0.036 |  |
| Mineral Mix S10026B | 67.5 |  | 67.5 |  | 67.5 |  | 67.5 |  |
| Vitamin Mix V10001C  (10X Concentrated) | 1.35 |  | 1.35 |  | 1.35 |  | 1.35 |  |
| Choline Bitartrate | 2.7 |  | 2.7 |  | 2.7 |  | 2.7 |  |
| Fat Fraction | 0 |  | 120 |  | 0 |  | 0 |  |
| Protein Fraction | 0 |  | 0 |  | 120 |  | 0 |  |
| WPPC | 0 |  | 0 |  | 0 |  | 120 |  |
| **Total grams**  **(Macronutrients)** | 1365.89 |  | 1382.39 |  | 1367.19 |  | 1378.59 |  |
|  | gm | kcal | gm | kcal | gm | kcal | gm | kcal |
| Protein | 322.5 | 1290 | 322.5 | 1290 | 322.5 | 1290 | 322.5 | 1290 |
| Carbohydrate | 591.5 | 2366 | 591.5 | 2366 | 591.5 | 2366 | 591.5 | 2366 |
| Fat | 188.7 | 1698 | 188.7 | 1698 | 188.7 | 1698 | 188.7 | 1698 |
| Total kcals |  | 5354 |  | 5354 |  | 5354 |  | 5354 |
| Kcal/gm |  | 3.9 |  | 3.9 |  | 3.9 |  | 3.9 |

| **Tissue type** | **Control** | **Protein** | **Fat** | **WPPC** |
| --- | --- | --- | --- | --- |
| **Liver (% of BW)** | 0.039±0.0031^a^ | 0.041±0.019 ^b^ | 0.043±0.0018 ^b^ | 0.041±.0014 ^a^ |
| **Spleen (% of BW)** | 0.088±0.014 ^a^ | 0.094±0.012 ^a^ | 0.099±0.013 ^a^ | 0.086±0.016 ^a^ |
| **iWAT (% of BW)** | 0.80±0.022 ^a^ | 0.57±0.12 ^b^ | 0.65±0.21 ^b^ | 0.73±0.22 ^a^ |
| **eWAT (% of BW)** | 1.4±0.36 ^a^ | 1.2±0.30 ^a^ | 1.4±0.28 ^a^ | 1.5±0.45 ^a^ |
| **BAT (% of BW)** | 0.16±0.030 ^a^ | 0.17±0.047 ^a^ | 0.18±0.016 ^a^ | 0.19±0.045 ^a^ |
| **Pancreas (% of BW)** | 0.20±0.012 ^a^ | 0.20±0.020 ^a^ | 0.19±0.024 ^a^ | 0.19±0.32 ^a^ |
| **Kidney (% of BW)** | 0.40±0.046 ^a^ | 0.42±0.045 ^a^ | 0.46±0.029 ^a^ | 0.43±0.050 ^a^ |
| **Colon (cm)** | 7.2±0.66 ^a^ | 7.3±0.46 ^a^ | 6.9±0.75 ^a^ | 6.1±0.53 ^a^ |
| **Small intestine (cm)** | 31.5±1.9 ^a^ | 33.1±1.7 ^a^ | 33.1±1.3 ^a^ | 32.3±0.51 ^a^ |

Supplementary Table 2: Organ and tissue weights and lengths.


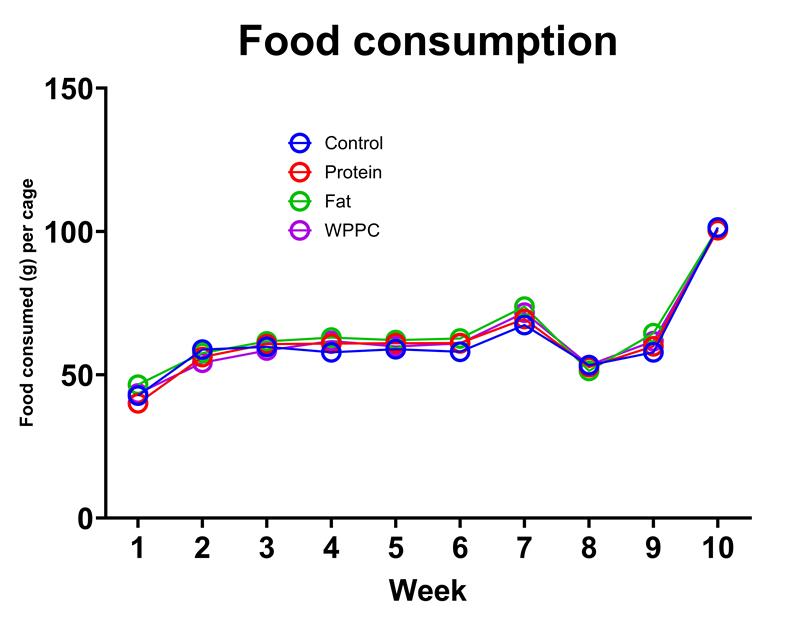

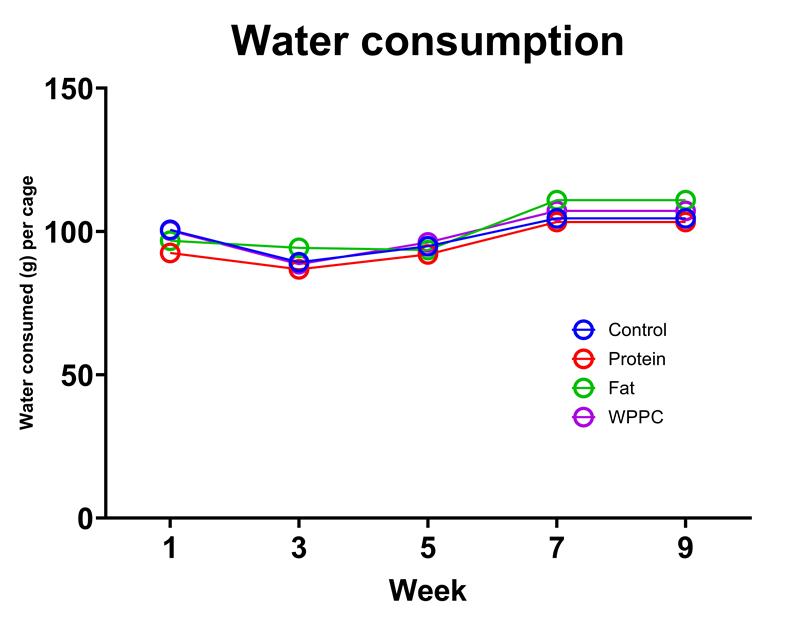


Supplementary figure 1: Food and water consumption


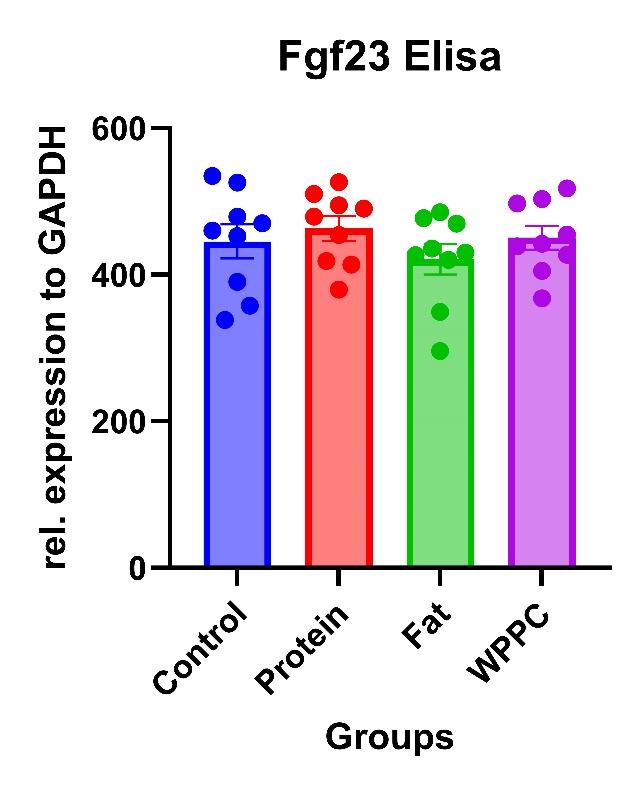


Supplementary Figure 2: Intact FGF23 serum levels.
